# Fuzzy logic control for watering system

**DOI:** 10.1038/s41598-023-45203-2

**Published:** 2023-10-28

**Authors:** Maciej Neugebauer, Cengiz Akdeniz, Vedat Demir, Hüseyin Yurdem

**Affiliations:** 1 Faculty of Technical Sciences, University of Warmia and Mazury in Olsztyn, Olsztyn, Poland Oczapowskiego, 10-719; 2 Ege University of Izmir, Campus 35100, Bornova, Izmir Turkey

**Keywords:** Environmental impact, Electrical and electronic engineering

## Abstract

A two-dimensional finite element (FEM) model was developed to simulate water propagation in soil during irrigation. The first dimension was water distribution depth in soil, and the second dimension was time. The developed model was tested by analyzing water distribution in a conventional (clock-controlled) irrigation model. The values in the conventional model were calculated based on the literature. The results were consistent with the results obtained from the model. In the next step, a fuzzy logic model for irrigation control was developed. The input variables were ambient temperature, soil moisture content and time of day (which is related to solar radiation and evapotranspiration), and the output variable was irrigation intensity. The fuzzy logic control (FLC) model was tested by simulating water distribution in soil and comparing water consumption in both models. The study demonstrated that the depth of the soil moisture sensor affected water use in the fuzzy logic-controlled irrigation system relative to the conventional model. Water consumption was reduced by around 12% when the soil moisture sensor was positioned at an optimal depth, but it increased by around 20% when sensor depth was not optimal. The extent to which the distribution of fuzzy variables affects irrigation performance was examined, and the analysis revealed that inadequate distribution of fuzzy variables in the irrigation control system can increase total water consumption by up to 38% relative to the conventional model. It can be concluded that a fuzzy logic-controlled irrigation system can reduce water consumption, but the system’s operating parameters should be always selected based on an analysis of local conditions to avoid an unintended increase in water use. A well-designed FLC can decrease water use in agriculture (thus contributing to rational management of scarce water resources), decrease energy consumption, and reduce the risk of crop pollution with contaminated groundwater.

## Introduction

Growing anthropogenic pressure has accelerated the risk of ecosystem damage at the beginning of the twenty-first century. Climate change, the energy crisis and environmental pollution are only some of the examples of the global human footprint. Some of these threats, such as increasing CO_2_ emissions and the rise in the average global temperature, have attracted considerable attention in the media. However the other problems are addressed only by small communities of experts, despite the fact that their consequences are equally dire. Agricultural drought belongs to the latter category of problems. Agriculture is facing a major crisis in the twenty-first century. The main challenges in the farming sector include the need to increase global food supply to feed a growing population, declining availability of soil and water resources, and high greenhouse gas emissions (such as methane) in livestock production.

Water deficit poses a significant problem in the farming sector. Agricultural drought decreases yields and farming profits, and it leads to an increase in global food prices. Various irrigation systems are applied to resolve this problem. Irrigation is the main source of water for crops in countries with a hot and arid climate, such as Turkey. In countries with a temperate climate, such as Poland, irrigation systems are built mainly in large farms and vegetable farms, including in greenhouse farms where irrigation is essential. However, due to progressive agricultural drought, irrigation systems are increasingly introduced in small farms and crop fields. Despite the above, irrigation cannot fully resolve the global water deficit problem. The declining availability of surface and underground water^1,2^ reduces the amount of water that can be utilized in irrigation systems. Therefore, rational water management, including in irrigation systems, is a very important consideration. In China, a fuzzy logic model was developed to select agricultural areas in the province of Gansu that require irrigation^3^. The Chinese model was developed on a much larger scale than that described in this study, but the proposed solution demonstrates that agricultural drought poses a severe threat and that water resources have to be rationally managed.

Irrigation systems are composed of water pipes, sprinklers and valves, which have been thoroughly investigated based on the principles of fluid mechanics and installation practices, leaving little room for improvement in the mechanical design of irrigation systems. However, modern technologies have considerable potential for controlling irrigation systems^4^.

At present, the majority of irrigation systems around the world are controlled manually. The system is activated manually in a selected area, water flow is switched to a different area after a given time, and the system is manually deactivated. These operations require time, which could pose a problem in large farms that are increasingly automated and employ fewer workers. Automatic irrigation systems have been designed to address these concerns. Irrigation systems where watering time is controlled by a clock have a simple design and are more reliable. The system is activated and deactivated automatically based on the programmed time schedule. The main advantage of a clock-controlled irrigation system is its simple structure which consists of water distribution pipes, a pump, a power source and a clock controller (timer). However, a clock-controlled system also operates when watering is not required, for example in rainy weather or when soil moisture content is high. The system has to be manually deactivated in rainy weather, and it has to be activated at an appropriate moment to prevent soil from drying after the rain.

Various types of automatic irrigation control systems have been designed, where the frequency and duration of watering is controlled automatically based on weather conditions, time of day and other parameters. Numerous irrigation systems have been described in the literature, beginning from the simplest clock-controlled systems to sophisticated solutions where irrigation is controlled by PID controllers based on physical parameters such as ambient temperature, soil moisture content and time of day. In recent years, IoT solutions have been used to design smart irrigation systems^5^. A fuzzy model was developed by Tamilvanan et al.^6^ to evaluate soil moisture content. According to the authors, the model can be applied in irrigation systems, and soil moisture content can be determined based on other physical parameters, such as ambient temperature and humidity.

However, PID controllers and similar solutions are binary devices that have only two states (0 or 1), and they are not always ideally suited for irrigation control. These devices are simple, and they do not have to be operated manually on a daily basis because system settings are based on long-term calculations. In some cases, however, digitally controlled systems can use more water that is needed by crops^7^. In order to minimize the risk of this event, it was decided to use one of the modern tools for controlling nonlinear complex systems. Examples of such tools include Netrosophic Logic or Statistic (NL or NS) and Fuzzy Loigic (FL)^8–10^. The main difference between them is that in the case of NS, the sums of the probabilities of individual variables may exceed 1, and for FL they must be exactly equal to 1^11^. Later in the work, it was decided to use FL to build FLC. An example of the difference between a classical control system and one based on fuzzy logic can be found in Patel and Shah^12^. This choice was dictated by the fact that the first author had a license for the FLC creation software—which is part of the LabView package. At the same time, in the literature you can find many works showing examples, often only in the theoretical description of the use of fuzzy logic controllers for example—Patel and Shah^13,14^. Binary devices are replaced with other solutions to minimize water consumption during irrigation, while effectively catering to the watering requirements of plants. Various control systems have been proposed in the literature, including solutions that are based on fuzzy logic. Different fuzzy logic controllers have been described, where the input variables include temperature only or temperature, humidity and solar radiation, and where watering time is the output variable. Most of the proposed systems are theoretical solutions that describe a fuzzy logic-controlled irrigation system (selection and description of input and output variables) and simulate the performance of a watering system. This type of solution was described by Kia et al.^15^. The advantage of systems based on fuzzy logic is that they can easily use human knowledge^16,17^—e.g. experts in a given field. Additionally, methods based on fuzzy logic were used, for example, to calibrate or estimate the size of the Hargreaves equations describing evapotranspiration^18^, which is one of the variables needed to calculate the water dose for plants^19,20^.

Practical implementations of the designed fuzzy logic controllers have been described in some studies^21,22^. However, the distribution of fuzzy input variables for irrigation systems has never been discussed in the literature. For example, the distribution of the linguistic value *cold* in the linguistic variable *temperature* was not explained or justified in the solution developed by Ref.^21^. In irrigation systems, ambient temperature is closely linked with the local climate and soil conditions. In a study by Al-Ali et al.^23^, the linguistic value *very hot* (35–60 °C) in the fuzzy variable *temperature* has a triangular membership function with a maximum at 50 °C, but such high temperatures are not encountered in many places in the world. Similar assumptions were made by Velado et al.^24^ or Benyezza et al.^25^. A fuzzy model was described in detail by Aboti et al.^26^, but the developed system was not tested in practice, and the modeled results were not compared with conventional systems. A fuzzy logic-controlled irrigation system for cereal production was designed in Qatar, where irrigation time was determined based on evapotranspiration data^27^.

However, there are works in the literature on the optimization of variables adopted for systems using fuzzy logic—but not directly related to the watering problem—compare^28,29^. Many fuzzy logic-controlled irrigation systems have been tested based only on the response of a fuzzy logic controller and fuzzy logic rules^30–32^. This approach was used by Khatri^33^ or Karaburun and Köse^34^ to evaluate the system’s applicability in a real-world setting and to compare its performance with conventional irrigation systems. Fuzzy logic controllers were simulated by Mohamad^35^ or Adak^36^, but the system’s performance was not tested in practice. In a study by Izzuddin et al.^37^, the entire irrigation system was modeled in Simulink, but soil conditions were not taken into consideration, and the proposed solution was not compared with conventional systems.

A fuzzy logic-controlled irrigation system was modeled in a two-step procedure by Truneh et al.^38^. In the first step, actual evapotranspiration was calculated based on physical data (humidity, temperature, net radiation and wind speed), and it was compared with desired evapotranspiration. The results of the comparison were used as input variables in a fuzzy logic-controlled irrigation system, and to calculate valve opening times. In the cited study, fuzzy logic-controlled irrigation systems were not compared with conventional watering systems. A simpler approach was adopted by Nandhini and AmudhaPrabha^39^ who did not calculate theoretical or actual evapotranspiration. However, the authors analyzed different watering systems: a sprinkler system and a drip irrigation system. A fuzzy logic-controlled irrigation system for roses was designed for a specific location in Mexico, but the designed solution was not compared with conventional watering systems^40^. A comprehensive irrigation system based on fuzzy logic was developed by Ref.^41^. The authors concluded that the proposed system considerably decreased water use, however, the amount of saved water was not given, and the relevant calculations were not presented.

Due to the general scarcity of published studies comparing conventional (clock-controlled) irrigation systems with fuzzy logic-controlled systems, this study was undertaken to model soil phenomena during watering to compare the performance of two irrigation systems. A theoretical model of a fuzzy logic-controlled irrigation system was developed to avoid high implementation costs. The modeled results were evaluated to determine whether the designed system outperforms conventional watering systems under specific soil and climatic conditions.

The developed model was applied to simulate the performance of a conventional (clock-controlled) irrigation system, where the amount of supplied water and watering intervals were calculated based on Good Agricultural Practices, and to evaluate the performance of a fuzzy logic-controlled irrigation system for different distributions of the same input variables. It was assumed that the modeled control system should be as simple as possible: it should feature the smallest possible number of sensors (to minimize the number of input variables), and it should operate locally without Internet access. These assumptions influenced the selection of input and output variables. The procedure of calculating vertical infiltration of water into the soil was presented by Macioszczyk^42^, and actual evapotranspiration was calculated based on the work of Lechnio^43^.

To the best of the authors’ knowledge, this is the first study where a model of water propagation in soil was developed to compare the performance of a fuzzy logic-controlled irrigation system with a conventional clock-controlled watering system.

The novelty of this work is the construction of a 2-D model of water propagation in the ground, which enables quick and cheap verification and comparison of different watering systems. This makes it possible to choose a watering system that, under given local conditions, will allow water to be supplied to the root zone of plants with the least amount of water consumption. The authors' contribution consisted in creating a model of water propagation in the ground, building a watering system using fuzzy control and practical verification of the obtained model and real watering systems. As written earlier, many authors analyse various control systems for watering plants, but their works lack reference to models and often no practical verification. The built model will also enable testing of other (not only fuzzy) control systems for watering systems. Another novelty is the finding that the depth of placement of the humidity sensor affects the amount of water used with sufficient irrigation of the plant root zone. Consequently, the proper selection of the depth of placement of the humidity sensor is important and should be determined for different types of soil and plants.

## Research aim and objectives

The aim of this study was to develop a model of water propagation in soil to evaluate the performance of a fuzzy logic-controlled irrigation system. Engineering practice dictates that simpler solutions are generally more effective and reliable. This assumption was adopted in the process of selecting input variables and their distribution.

The objectives of this study were to:

- Describe the main phenomena associated with water propagation in soil during irrigation;
- Build a two-dimensional model based on the finite element method (FEM) to describe water propagation in soil over time;
- Propose watering guidelines for specific soil conditions and crop species;
- Design a fuzzy logic-controlled irrigation system and analyze the relationships between input and output variables;
- Test and compare the performance of conventional and fuzzy logic-controlled irrigation systems under specific soil conditions.

## Materials and methods

**Figure 1:**
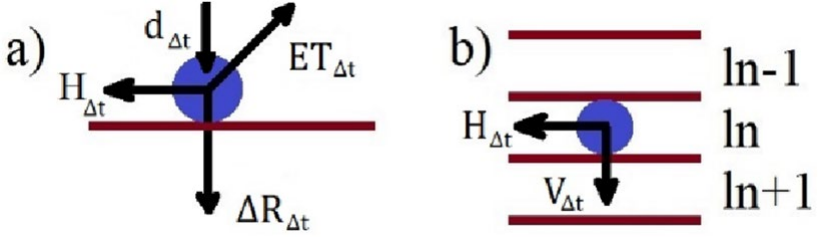
Water behavior at every time interval *∆t*: (**a**) on soil surface: d_∆t_—distance traveled by water, H_∆t_—runoff, ∆R_∆t_—infiltration/vertical gravity flow, ET_∆t_—evapotranspiration; (**b**) in the nth soil layer: H_∆t_—capillary (horizontal) infiltration, V_∆t_—gravity flow (vertical). Source: own elaboration.

The main phenomena associated with water propagation in soil were described and presented in Fig. 1. The initial assumption was that every unit area of irrigated soil is supplied with the same amount of water (calculated based on sprinkler parameters). The general principles for modeling and controlling irrigation systems were described by Angelaki et al.^44^. Various methods for modeling soil moisture based on environmental conditions, including methods that rely on artificial neural networks, were presented by Dewidar et al.^45^ or Alekseev and Vasilyev^46^.

Surface water flow was determined with the use of the Penck-Oppokov equation:

1 $$H= d - ET - \Delta R,$$

where: *H* – horizontal runoff [mm], *d*—distance traveled by water [mm], *ET*—evapotranspiration [mm], *∆R*—infiltration/ vertical gravity flow [mm].

Groundwater flow in the nth soil horizon was determined with the use of Darcy’s law:

2 $$v \, = \, k \cdot i.$$

Water velocity *v* was converted to actual water velocity *v*_*s*_ with the use of the continuity equation:

3 $${v}_{s}=\frac{v}{n}=\frac{ki}{n},$$

where: *v*—ater velocity [m⋅s^−1^], *v*_*s*_—actual water velocity in the ground [m⋅s^−1^], *k*—filtration coefficient [m⋅s^−1^], *i*—hydraulic gradient (gradient) [−], *n*—porosity [−].

A two-dimensional model of water behavior in soil at time ∆t was developed with the use of the FEM and Eqs. (1), (2) and (3) (after unit conversion). The first dimension was vertical water flow in soil, and the second dimension was time. Based on a review of the literature, the mesh size of the FEM model was set at 288 × 140.

Differential equations with time step ∆t = 1 h were derived and solved in the Stella program, and the results were visualized in Excel spreadsheets. The filtration coefficient *k* and the developed model were verified based on the results of an unpublished study^47^.

Evapotranspiration was calculated with the use of the formula presented by Cetin and Beyhan^48^ based on real-world temperature and solar radiation measured in Izmir, Turkey, in September 2021.

The model of a conventional irrigation system was developed for the soil and plant parameters presented in Table 1. Saturated conductivity (steady infiltration rates) was set at 13 mm/h, which corresponds to loam conditions.

**Table 1:** Soil and plant parameters.

Soil parameters			
Soil type	Loam		
Bulk density (weight per unit volume)	*ρb*	1.36	kg dm^−3^
Soil infiltration rate	*I*	13	mm h^−1^
Field capacity	*FC*	22	%
Permanent wilting point	*WP*	10	%
Plant parameters			
Plant	Grass		
Effective root zone depth	*Drz*	0.2	m
Management allowed deficit	*MAD*	50	%
Evapotranspiration (daily water consumption)	*ET*	12	mm day^−1^
Shading percentage	*Pd*	100	%
Maximum evaporation rate	*Tr*	1	
Application uniformity	*Ea*	40	%
Water density	*ρw*	1	kg dm^−3^
Sprinkler application rate	*Is*	9	mm⋅h^−1^

Based on the above data, irrigation time and water use were calculated with the use of formulas (4), (5), (6), (7), (8), (9), (10) and (11), because the actual research will be carried out in field conditions—formulas used to determine the required dosage of water ware taken among others from Finkel^49^, Benami and Ofen^50^, Keller and Bliesner^51^:

4 $${\text{AWC}} = \left( {\left( {{\text{FC}} - {\text{WP}}} \right)/{1}00} \right) \cdot ({\text{rb}}/{\text{rw}}) \, = { 163}.{\text{2 mm m}}^{{ - {1}}} ,$$

5 $${\text{dn}} = {\text{AWC}} \cdot {\text{Drz}} \cdot {\text{MAD }} = { 16}.{\text{32 mm}},$$

6 $${\text{Etd}} = {\text{ ET}} \cdot 0.{1} \cdot \left( {{\text{Pd}}} \right)^{{0.{5}}} = {\text{ 12 mm day}}^{{ - {1}}} ,$$

7 $${\text{Ti}} = {\text{dn}}/{\text{Etd }} = { 1}.{\text{36 day}},{\text{ where Ti}}^{\prime} \approx {\text{1 day}},$$

8 $${\text{d}} = {\text{Etd}}*{\text{Ti}}^{\prime} \, = { 15}.{\text{6 mm}},$$

9 $${\text{dt}} = {\text{d}}*{\text{Tr}}/{\text{Ea }} = {\text{ 39 mm}},$$

10 $${\text{ndw}} = {\text{ET}}/{\text{Ea }} = { 3}0{\text{ mm day}}^{{ - {1}}} ,$$

11 $${\text{Ta}} = {\text{dt}}/{\text{Is }} = { 4}.{33} \approx {4}.{\text{5 h}},$$

where: *AWC*—available water-holding capacity, *dn*—net irrigation depth per treatment, *Etd*—daily irrigation water use, *Ti*—irrigation interval, *d*—demand for irrigation water in selected intervals, *dt*—total irrigation depth per treatment, *ndw*—net daily water use, *Ta*—irrigation time.

Sprinklers with an application rate of 9 mm·h^−1^ were operated for around 4.5 h per day in the conventional irrigation system.

A fuzzy logic controller for the irrigation system was developed in the next stage of the study. The input variables were ambient temperature, soil moisture and time of day. The number of variables was kept to a minimum to simplify the structure of the irrigation system in a real-world setting. Temperature was selected as the input variable because it affects the evaporation rate of water (not evapotranspiration which was included in the model of water propagation in soil). Temperature distribution was based on real-world measurements conducted in Izmir, Turkey, in the first 2 weeks of September. Evaporation rate is also influenced by solar radiation, but this parameter was expressed by the time of day in the fuzzy logic model. Time of day was determined based on clock time. Soil moisture (described as *sensor data* in Table 3) will be measured at a specific depth in a real-world irrigation system.

**Figure 2:**
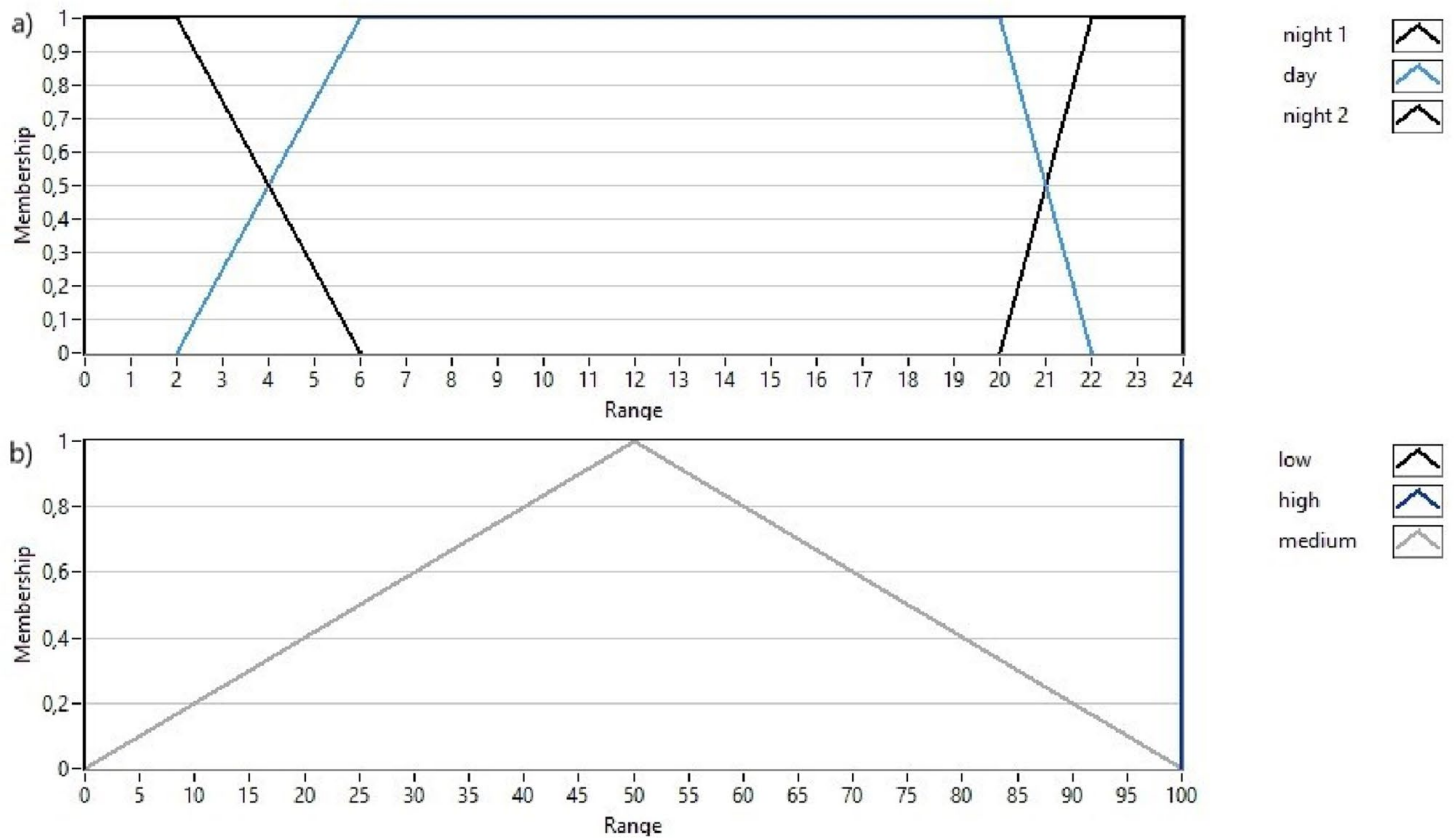
Distribution of *time of day* input variable (**a**) and *irrigation intensity* output variable (**b**).

**Figure 3:**
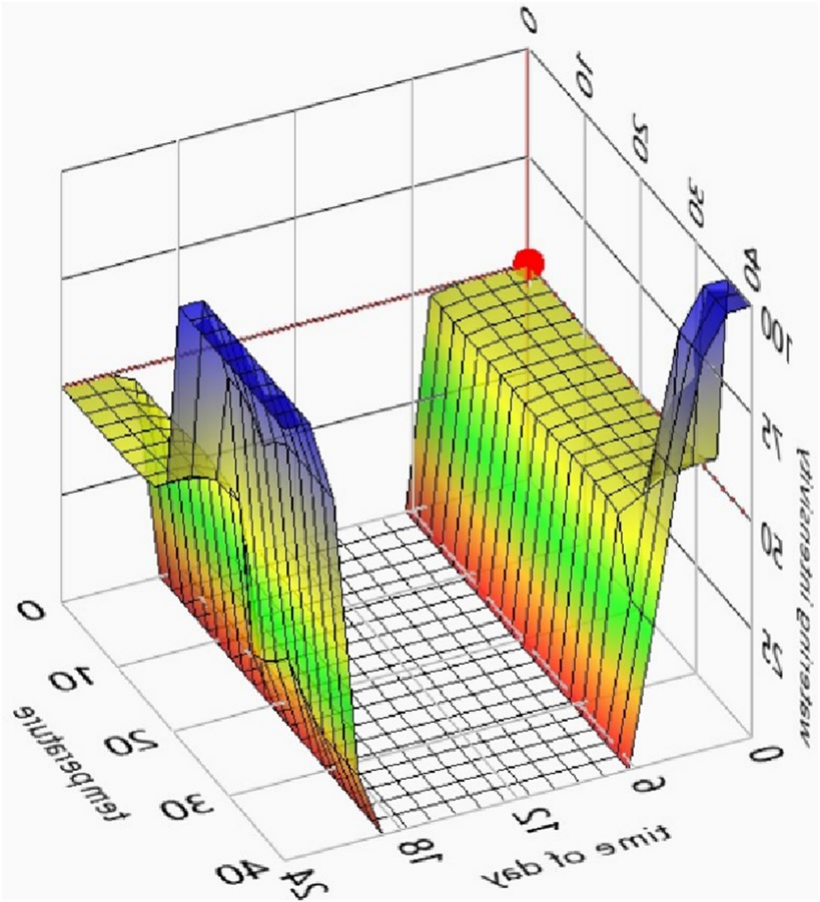
Response of the designed fuzzy logic controller.

The output variable was the pumping flow rate, where the calculated maximum hourly sprinkler application rate (formulas (4), (5), (6), (7), (8), (9), (10) and (11) was adopted as 100%. The distribution of the input variable (*time of day*) and the output variable in preliminary simulations is presented in Fig. 2a and b. The distribution of terms for variables was made using the knowledge of specialists in the field of watering, in accordance with what is given in the literature, fuzzy logic systems are very suitable for this. However, error tolerance was not tested in the adopted fuzzy controller—compare, for example, Patel and Shah^52^. The variables are described in detail in Table 2. The values in column A were used in simulations (a) and (b), and the values in column B were used in simulation (c) (described below). The fuzzy rules for each set of variables are presented in Table 3. The output variable was calculated for the central point in the irrigated area. The relationships between input variables (*temperature* and *time of day*) and irrigation intensity are presented in Fig. 3. The fuzzy model was developed in the LabView environment to facilitate future application in the designed system.

**Table 2:** Distribution of input and output variables in the fuzzy model.

Variables	Membership function	Shape	Points	Membership function	Shape	Points
Time of day	Night	Trapezoid	0; 0; 4; 8	Night	Trapezoid	0; 0; 2; 6
	Day	Trapezoid	4; 8; 18; 22	Day	Trapezoid	2; 6; 20; 22
	Night	Trapezoid	18; 22; 24; 24	Night	Trapezoid	20; 22; 24; 24
Soil moisture	Dry	Trapezoid	0; 0; 30; 75	Dry	Trapezoid	0; 0; 5; 50
	Wet	Trapezoid	25; 75; 100; 100	Wet	Trapezoid	5; 50; 100; 100
Temperature	Cold	Trapezoid	0; 0; 10; 20	Cold	Trapezoid	0; 0; 5; 20
	Moderate	Triangle	10; 20; 30	Moderate	Triangle	5; 20; 35
	Hot	Trapezoid	20; 30; 40; 40	Hot	Trapezoid	20; 35; 40; 40
Irrigation intensity	Low	Singleton	0	Low	Singleton	0
	High	Singleton	100	High	Singleton	100
	Medium	Triangle	0; 50; 100	Medium	Triangle	0; 50; 100

A—values for simulations (a) and (b). B—values for simulation (c).

**Table 3:** Fuzzy control rules.

	Input			Output
	Time of day	Sensor data	Temperature	Irrigation intensity
1	Night	Dry	Cold	Medium
2	Night	Dry	Moderate	Medium
3	Night	Dry	Hot	High
4	Night	Wet	Cold	Low
5	Night	Wet	Moderate	Low
6	Night	Wet	Hot	Low
7	Day	Dry	Cold	Low
8	Day	Dry	Moderate	Low
9	Day	Dry	Hot	Low
10	Day	Wet	Cold	Low
11	day	wet	moderate	Low
12	Day	Wet	Hot	Low
13	Night	Dry	Cold	Medium
14	Night	Dry	Moderate	High
15	Night	Dry	Hot	High
16	Night	Wet	Cold	Low
17	Night	Wet	Moderate	Low
18	Night	Wet	Hot	Low

The irrigation system was simulated in three variants:

- The soil moisture sensor was positioned at nine different depths in the fuzzy logic-controlled irrigation system;
- The depth of the soil moisture sensor was determined based on the results of a previous study for the ambient temperatures measured on 1 October 2021;
- Different distribution of input variables.

**Figure 4:**
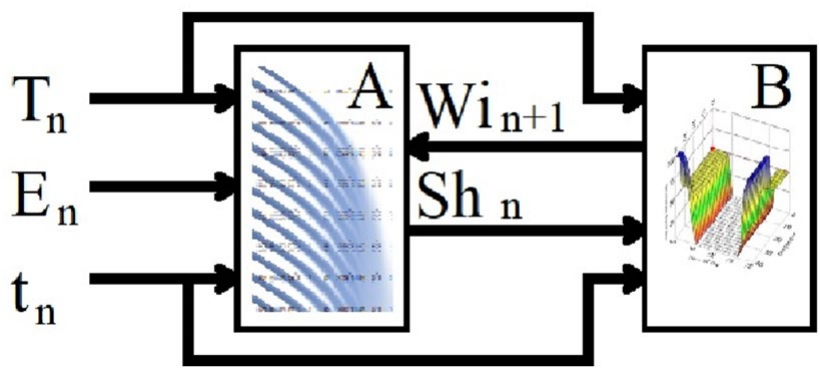
Diagram of data flow during the simulation. (**A**) model of water propagation in soil, (**B**) model of a fuzzy-logic controlled irrigation system; *Tn* temperature at time *n*, *En* evapotranspiration at time *n*; *tn* hour, *Sh* soil moisture at time *n*, *Wi* irrigation intensity at time *n* + *1*.

Soil moisture at every time *t* was calculated in the model and was used as the input variable in the irrigation control model (Fig. 4).

## Results and discussion

The starting point for the models was selected on the assumption that soil was completely dry across the entire simulated depth. The simulation covered a period of 12 days. All of the tested models achieved convergence during that time because temperature distribution was identical on each modeled day. Identical temperature distribution was adopted to ensure that simulation models begin to repeat after a specified time, depending on sensor depth. This moment was regarded as the moment at which the model achieved convergence with real-world data (water propagation in soil and soil saturation).

**Figure 5:**
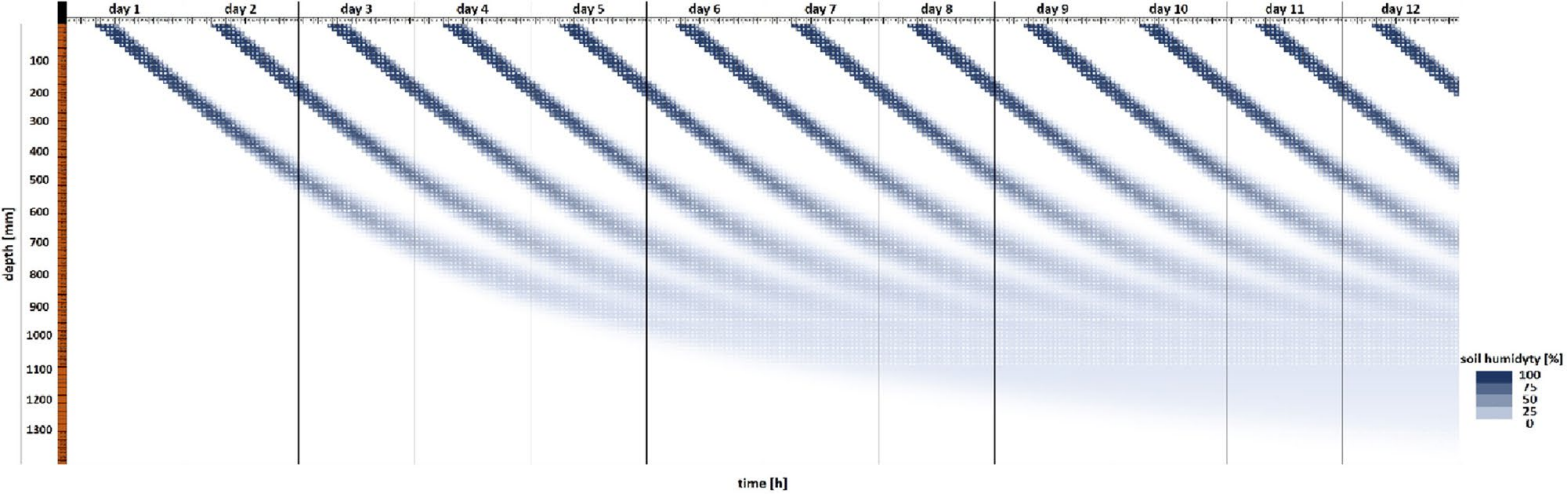
FEM model simulating water propagation as a function of irrigation depth and time based on the values calculated with the use of formulas (4), (5), (6), (7), (8), (9), (10) and (11).

**Figure 6:**
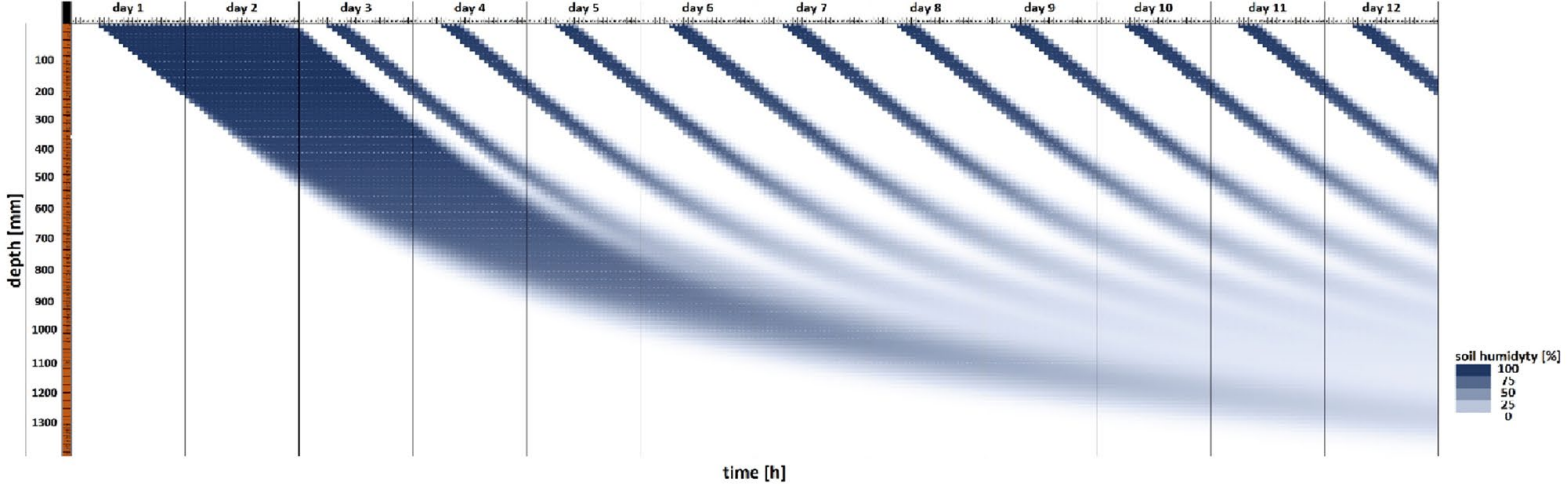
FEM model simulating water propagation as a function of irrigation depth and time based on the values calculated with the use of formulas (4), (5), (6), (7), (8), (9), (10) and (11) when water use on the first day of the irrigation interval was three times higher than the calculated value.

Water propagation over time (conventional control system) was simulated in the FEM model for longer irrigation times and higher water use (Fig. 5). A comparison of *dt* values calculated with the use of formula (9) in the conventional system after 4.5 h of irrigation (Fig. 5) indicates that the model is consistent with the calculations. The behavior of the model when water consumption on the first day of the irrigation interval was three times higher than the calculated value (formulas (4), (5), (6), (7), (8), (9), (10) and (11)) is presented in Fig. 6.

**Figure 7:**
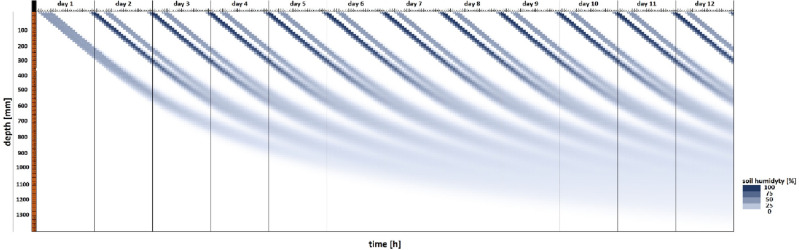
Distribution of water propagation in soil over time in a fuzzy logic-controlled irrigation system. The simulation was conducted for sensor depth of 30 mm.

The distribution of water propagation in soil (Fuzzy logic control system) at one of the tested sensor depths is presented in Fig. 7.

**Figure 8:**
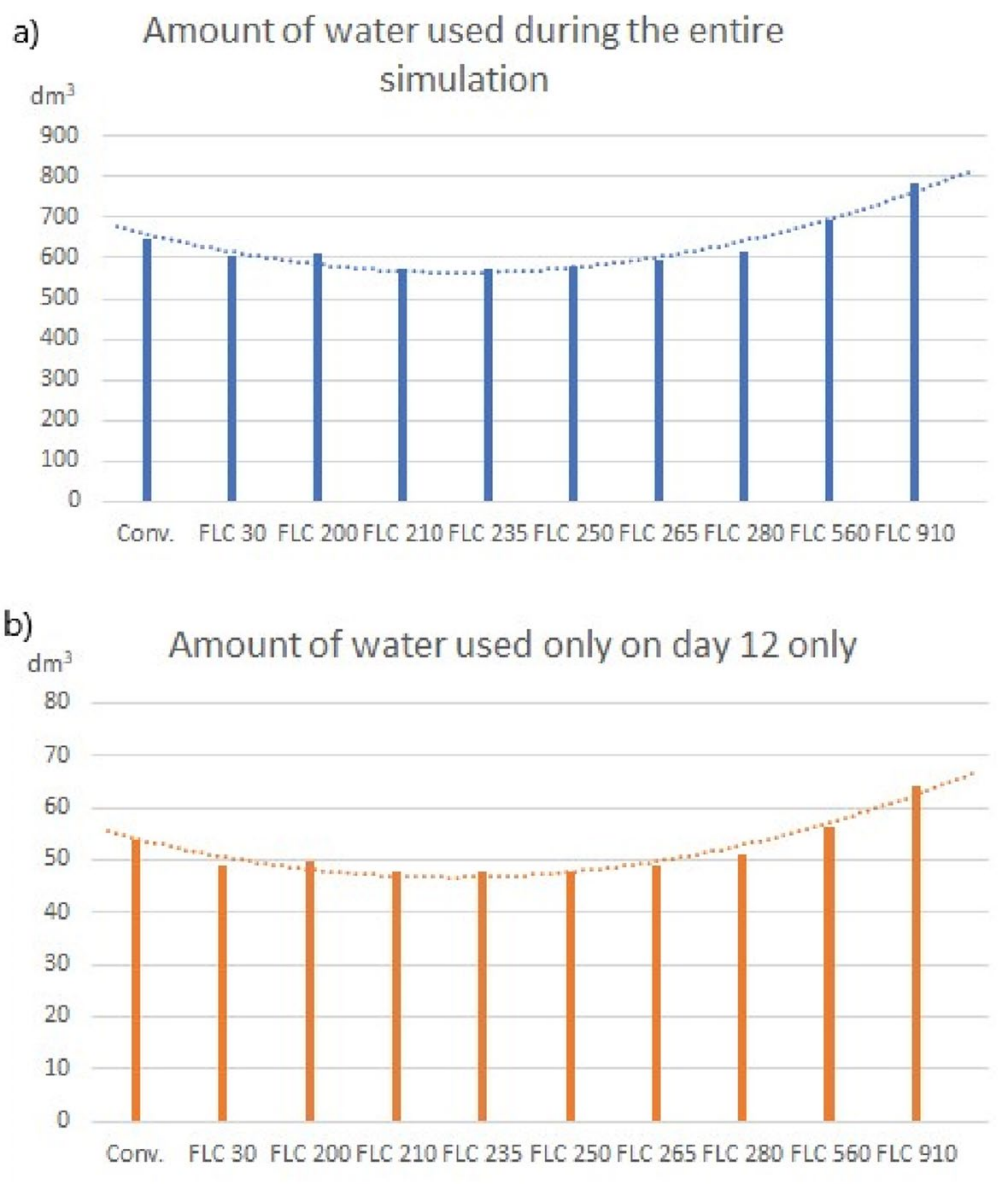
Amount of water used during the entire simulation and on day 12. Conventional (conv.)—clock-controlled irrigation system; FLC—fuzzy logic-controlled irrigation system. Numbers after the letters FLC indicate sensor placement depth [mm]. (**a**) for the entire simulation; (**b**) for the 12 day only.

The results of simulation (a) will be discussed first. Total water consumption differed at various sensor depths for the same distribution of the remaining values. Water use at different sensor depths in each of the analyzed variants for the entire simulation (12 days) and for the last day (when the model achieved convergence) is presented in Fig. 8.

The results clearly indicate that sensor depth significantly influenced the performance of the irrigation system (lower water use relative to the conventional irrigation system). To the best of the authors’ knowledge, this is the first study to report on the correlation between sensor depth and irrigation performance. Water use was lowest (573.1 dm^3^ and 47.6 dm^3^ for the entire simulation and for day 12, respectively) when the moisture sensor was placed at a depth of 235 mm. In this scenario, water use was reduced by 11.5% and 12%, respectively, relative to the conventional irrigation system.

## Comparison between FLC and conventional controller

**Figure 9:**
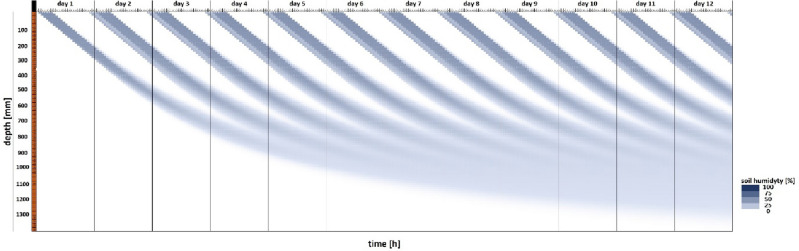
Distribution of water propagation in soil in the fuzzy logic-controlled irrigation system. Simulation for sensor depth of 235 mm and real-world temperatures measured on 1 October 2021.

In the next step, the performance of conventional and fuzzy logic-controlled irrigation systems was simulated for a different distribution of temperature values based on the real-world measurements conducted in Izmir on 1 October 2021 (for sensor depth of 235 mm). The results are presented in Fig. 9. Total water use decreased to 559.5 dm^3^ for the entire simulation and to 46.6 dm^3^ on day 12. In both cases, the fuzzy logic-controlled system used 13% less water than the conventional system.

The next simulation involved a different distribution of input and output variables in the fuzzy logic-controlled system, but with the same fuzzy logic rules (the changes are presented in column B in Table 2). The calculated water use was 894.6 dm^3^. Water use increased by more than 38% in both cases relative to the conventional system (where the analyzed parameter did not change).

The conducted simulations clearly indicate that the distribution of variables in a fuzzy logic-controlled system significantly affects water use. To the best of the authors’ knowledge, these correlations have never been analyzed in the literature. The distribution of input variables should be carefully selected and the optimal water dose should be calculated based on the performance of the conventional irrigation system (for example, with the use of formulas (4), (5), (6), (7), (8), (9), (10) and (11)) because these data constitute a reliable reference point for simulating the performance of a fuzzy logic-controlled irrigation system. This approach should be adopted to maximize the performance and reduce water use in a fuzzy logic-controlled watering system. Lower water consumption will also decrease energy requirements for powering the water pump or another irrigation system. In Turkey, groundwater is strongly polluted with nitrates^53^, and surface water can reach groundwater when irrigation is abundant. The capillary movement of soil water increases the risk that pollutants will reach the effective root zone, which can negatively affect crop growth. Reduced water use (but sufficient to meet crop requirements) minimizes that risk, which is yet another advantage of a well-designed fuzzy logic-controlled irrigation system.

## Summary and conclusions

The basic assumption of the work was to develop a plant watering control system that would reduce water consumption and at the same time maintain the full humidity required by the plants in the root zone. The potential application of the developed system is primarily in crops grown in warm climates with little rainfall—e.g. in Turkey. At the same time, due to the constantly deepening water problems, an example is the agricultural drought that has been deepening in Poland for years—control systems for watering plants in fields, minimizing the consumption of groundwater while maintaining full yields, can also be used, for example, in Poland. The performance of the designed fuzzy logic-controlled irrigation system was validated with the use of a simulation model, which is standard practice in the process of developing new engineering solutions. The proposed solution is first validated through simulation, and if the results are promising or positive, the developed concept is tested in a laboratory prototype. This approach minimizes the cost and time needed to validate concepts that will not work well in practice.

The described model of water propagation in soil during irrigation was developed with the use of the FEM in two-dimensional space (sensor depth and time) to validate the performance of a fuzzy logic controller in the irrigation process. The system’s performance was assessed based on water consumption and watering time for a selected plant species (grass) with specific root depth. Soil parameters were calculated with the use of standard formulas. The developed model was used to test a conventional and a fuzzy logic-controlled irrigation system. The model assumptions were identical in both variants; therefore, the results of the simulation could be reliably compared in different irrigation control systems. A well-designed FLC can decrease water consumption in plant production (thus contributing to sustainable management of scarce water resources), decrease energy use and minimize the flow of polluted groundwater to the root zone of agricultural plants.

The following conclusions can be formulated based on the presented results:

- A well-designed model effectively depicts changes in external conditions, such as increased water use during irrigation or changes in ambient temperature;
- The depth of the soil moisture sensor (one of the variables in the FLC model) influences total consumption of irrigation water. When the position of the moisture sensor was too deep or too shallow, water consumption was higher in the simulated system than in the conventional system (clock-controlled system, where water use was calculated theoretically). When the sensor was placed at a depth of 910 mm, water consumption was approximately 20% higher in the fuzzy logic-controlled system (782.3 dm^3^ and 64.2 dm^3^, respectively) than in the conventional system;
- When sensor depth was optimal (235 mm), water use was lower (by 11.5% and 12%, respectively) in the fuzzy logic-controlled system than in the conventional system during the entire 12-day simulation period and on the last day of the simulation;
- When physical parameters are constant, the selection of input variables in a fuzzy logic-controlled irrigation system significantly affects water use. Therefore, the distribution of input and output variables in a fuzzy logic-controlled system should be carefully designed to minimize the risk of an opposite outcome (higher water consumption). In this study, changes in the modeled variables increased water use by around 38% in the fuzzy logic-controlled system relative to the conventional irrigation system.

In the future, the fuzzy logic-controlled irrigation system will be tested in the laboratory. If the above results and conclusions are empirically validated and if the fuzzy logic controller effectively reduces water consumption under laboratory conditions, the designed system will be tested under field conditions. In particular, attention should be paid to and examined what impact the adopted distribution of variable terms has on the functioning of the actual system. As part of this work, the distribution of variable terms was not optimized, which should be done in subsequent studies. It is also necessary to examine the repeatability and reproducibility of the control system for various sensors, sprinklers, etc. It is also necessary to examine whether for various plants—the proposed solution will actually contribute to reducing water consumption while maintaining full yield. The developed model of water propagation in soil during irrigation will be thoroughly analysed in laboratory and field experiments.

## Data Availability

Data used for research and simulation come from measurements made during the internship. Raw data—graphic files of photos showing the speed of water propagation in the ground are available from the authors, while the rest is simulation data, numerous according to the formulas contained in the article. Individual data and calculations—in the form of tables available from the authors.
